# An Audit of UK Hospital Doctors’ Knowledge and Experience of Myalgic Encephalomyelitis

**DOI:** 10.3390/medicina57090885

**Published:** 2021-08-27

**Authors:** Keng Ngee Hng, Keith Geraghty, Derek F. H. Pheby

**Affiliations:** 1 ST7 General Internal Medicine and Gastroenterology (Ret), Doctors with M.E., Office 7, 37-39 Shakespeare Street, Southport PR8 5AB, UK; 2 Centre for Primary Care, Division of Population Health, Health Services Research and Primary Care, University of Manchester, Manchester M13 9PL, UK; keithgeraghty2@gmail.com; 3 Society and Health, Buckinghamshire New University, High Wycombe HP11 2JZ, UK; derekpheby@btinternet.com

**Keywords:** myalgic encephalomyelitis, chronic fatigue syndrome, ME/CFS, ME, medical education, postgraduate education

## Abstract

*Background and Objectives:* There is some evidence that knowledge and understanding of ME among doctors is limited. Consequently, an audit study was carried out on a group of hospital doctors attending a training event to establish how much they knew about ME and their attitudes towards it. *Materials and Methods:* Participants at the training event were asked to complete a questionnaire, enquiring about prior knowledge and experience of ME and their approaches to diagnosis and treatment. A total of 44 completed questionnaires were returned. Responses were tabulated, proportions selecting available options determined, 95% confidence limits calculated, and the significance of associations determined by Fisher’s exact test. *Results:* Few respondents had any formal teaching on ME, though most had some experience of it. Few knew how to diagnose it and most lacked confidence in managing it. None of the respondents who had had teaching or prior experience of ME considered it a purely physical illness. Overall, 82% of participants believed ME was at least in part psychological. Most participants responded correctly to a series of propositions about the general epidemiology and chronicity of ME. There was little knowledge of definitions of ME, diagnosis, or of clinical manifestations. Understanding about appropriate management was very deficient. Similarly, there was little appreciation of the impact of the disease on daily living or quality of life. Where some doctors expressed confidence diagnosing or managing ME, this was misplaced as they were incorrect on the nature of ME, its diagnostic criteria and its treatment. *Conclusion:* This audit demonstrates that most doctors lack training and clinical expertise in ME. Nevertheless, participants recognised a need for further training and indicated a wish to participate in this. It is strongly recommended that factually correct and up-to-date medical education on ME be made a priority at undergraduate and postgraduate levels. It is also recommended that this audit be repeated following a period of medical education.

## 1. Introduction

Myalgic encephalomyelitis/chronic fatigue syndrome (ME/CFS) is a complex, multi-system illness defined by its clinical characteristics rather than by its underlying pathology, which remains obscure. These characteristics include severe incapacitating fatigue, post exertional malaise and other symptoms including cognitive dysfunction, orthostatic intolerance, muscle pain and sleep disturbances, with substantial reductions in functional activity and quality of life [1]. The severity, clinical course and duration of the illness are very variable. It most frequently occurs in the 20–50 age group and is more common in women than in men [2,3,4]. It is frequently asserted that there are some 250,000 sufferers in the UK [5]. If this is correct, there may be in the region of two million patients across Europe and over one million sufferers in the US [6].

A major problem faced by patients with ME/CFS is that many doctors do not recognise the condition as a genuine clinical entity. Disbelief is widespread, and many doctors lack knowledge and understanding of the illness. A recent literature review found that between a third and a half of GPs refused to accept the reality of the condition, that a similar proportion of patients were dissatisfied by the quality of primary care that they had received, and that similar proportions were reported across various geographical locations and had changed little over many years [7]. A study of the perceptions of European ME/CFS specialists concerning GP knowledge and understanding of the illness demonstrated serious misgivings about shortcomings, widely across Europe [8], and this is confirmed by a German paper that reported low satisfaction with medical care and that patients with ME/CFS are medically underserved [9]. It is also consistent with reports that individuals with ME/CFS in the US are medically underserved [10]. It has been argued that ME patients suffer delegitimation of their illness experience through their condition being defined as nonexistent or psychosomatic, leading to their being shamed or stigmatised as having a psychological disorder [11]. A US survey of emergency department attenders with ME/CFS found that 42% of such attenders were dismissed as having psychosomatic problems, and that staff lacked knowledge of the condition [12], while another American survey of patients with ME/CFS and other diseases of the neuro-endocrine-immune system including fibromyalgia and chronic Lyme disease found that 54.4% of respondents reported dissatisfaction with their medical care due to lack of training on the part of their physicians. In total, 71% consulted four or more physicians, and 63% took at least two years, before receiving a correct diagnosis, indicating a need for more education about these conditions in medical school, and for multi-system disease specialty clinics [13].

In this paper, the term ME (myalgic encephalomyelitis) is used in reporting our research findings, rather than the more usual ME/CFS, because that was the term used in the original training session on which this report is based. The term ME/CFS is used in reporting the relevant research literature, as the two terms are effectively synonymous.

As outlined above, it has long been the experience of patients with myalgic encephalomyelitis (ME) that their doctors have little knowledge and understanding of the condition and are largely unable to help. Worse, many report that their doctors do not believe their illness is real, resulting in lack of medical support. Examination of sample medical curricula in 2018 in the UK confirmed that ME was not in the syllabus at either undergraduate or postgraduate levels, and this is consistent with a report demonstrating serious inadequacies in undergraduate teaching about ME/CFS, in which 64% of responding medical schools acknowledged the need for improvement [14], and also with an earlier report from the US in which only 28% of responding medical schools met an adequate standard of coverage in their curricula [15].

It is therefore quite conceivable that patients’ widely reported impressions are well founded, so to investigate this, we undertook an ad hoc opportunistic audit of hospital doctors’ knowledge and understanding. This study appears to be the first attempt in the United Kingdom to assess knowledge and understanding of ME among a group of hospital doctors.

## 2. Methods

In 2018, we conducted an audit of hospital doctors attending a training event. Traditionally, response rates from physician-knowledge surveys are often low. As such, approaching doctors in person presented an informal setting and rapid way to gather responses.

All physicians in the region who were training in general internal medicine at ST3-8 level were required to attend this mandatory training day. Only those who were on-call or on leave would have been excused. There were in the region of one hundred attendees. Most of these GIM trainees were also training in another medical specialty, such as cardiology, respiratory medicine, endocrinology, nephrology, gastroenterology, neurology, rheumatology, haematology, dermatology, infectious diseases, palliative care, oncology, geriatrics or acute medicine.

This particular training day was unique in that a short introductory lecture on ME was scheduled. Other lectures were on unrelated topics. We developed a pre-planned questionnaire with input from experts in the field (Appendix A). These were handed out and returned on the same day. It was specified that answers should be based on participants’ knowledge before the lecture on ME. The questionnaire asked about prior knowledge and experience of ME, including previous education, confidence in managing the condition, and understanding of its epidemiology and pattern of chronicity. It also enquired about participants’ approaches to diagnosis and management, the perceived impact of the illness, and whether or not participants were interested in having additional education on ME.

A total of 44 completed questionnaires were returned. Responses were tabulated, proportions selecting available options determined, and 95% confidence limits calculated. Where relevant, associations between responses were presented in 2 × 2 tables, and the significance of such associations determined by Fisher’s exact test.

## 3. Results

### 3.1. Prior Teaching and Experience of ME, Doctors’ Confidence

Only 27% of respondents reported having previously received formal teaching on ME. Most of this was in the form of undergraduate or postgraduate lectures. 70% reported having had some experience of ME patients. This was in GP clinics, specialty clinics, or in hospitals. Twenty-three percent had had neither formal teaching on ME nor any experience of it.

A total of 89% of respondents admitted not knowing how to diagnose ME, which is very unsatisfactory. 93% did not feel confident dealing with ME patients. Only two respondents (5%) said they knew how to diagnose ME and also felt confident managing ME patients. However, one of them annotated “ish” against the answers indicating he/she was not fully confident, and the other annotated “If by ME chronic fatigue syndrome is meant,” indicating he/she did not understand the difference between the terms. These results are summarised in Table 1.

There was a significant association between being confident about diagnosing ME and feeling confident about dealing with ME patients (*p* = 0.029). These results are summarised in Table 2.

Doctors’ confidence was cross tabulated against key indicators of understanding, diagnostic ability and management. Of the six respondents who felt they knew how to diagnose ME or felt confident dealing with ME patients (i.e., five who said they knew how to diagnose ME and three who said they felt confident dealing with ME patients, or six in total as two were confident in both), all thought ME was partly or wholly psychological, and none selected the right combination of diagnostic criteria. All thought ME could be treated with graded exercise therapy (GET) and four thought it could be treated with cognitive behavioural therapy (CBT) to help patients get out of the sick role. Therefore, it appears that the greater the doctor’s confidence, the worse was his or her understanding of the illness and diagnostic skill. These observations are interesting, though they do not reach statistical significance since numbers were small.

On the central question of whether or not ME was thought to be entirely or in part a psychological or psychosomatic illness, respondents were given the options of psychological/psychosomatic or physical illness, and they were allowed to tick both (i.e., with a substantial psychological element). The correct answer, selected by only four respondents (9.1%), was a physical illness only, while 36 out of the 44 respondents (81.8%) believed ME was partly or entirely psychological.

The responses regarding whether participants had received prior teaching on ME or had seen ME patients were cross tabulated against responses to the question as to whether ME was thought to be a physical illness or at least in part psychological. All four respondents who understood that ME is a real, physical illness are among the ten who had received no formal teaching on ME, nor ever seen any ME patients (i.e., 40%), compared to 0% of respondents who had received previous formal teaching on ME or had seen any ME patients (Table 3). This was a very strong association (*p* = 0.0015). This begs the question as to what they were being taught on ME, and what they were told by their colleagues when they came across ME patients in the clinical setting.

### 3.2. General Epidemiology

Respondents performed fairly well on questions relating to the general epidemiology and chronicity of the illness. A series of propositions were put to respondents, who were asked to identify whether they were true or false. Correct responses ranged from 56.8% to 97.7% (average 82.3%). However, it was a matter of some concern that around a third of respondents considered the statement “children with ME miss school because their parents support their sick role and this should be discouraged” to be correct (Table 4).

### 3.3. Definitions and Clinical Understanding

Respondents performed poorly on overall categorisation, with 66% of them wrongly believing that ME belonged in the class of illness called medically unexplained symptoms and 59% of them not knowing the difference between ME, chronic fatigue syndrome and post viral fatigue syndrome. On the manifestations and impact of the illness, there was widespread appreciation that the illness was painful, but it was not generally appreciated that ME could affect all body systems and could be lethal. Nor was it appreciated that ME can be severely disabling. The approach to management was equally misguided, with only two respondents (4.5%) disagreeing with the false proposition that “patients need to think positive and build up their strength with exercise or gradually increasing activity.” (Table 5).

### 3.4. Diagnostic Process and Diagnostic Criteria

Respondents were asked which part of the process was the most important in making a diagnosis of ME, which is a careful history. In answer to “ME is mainly diagnosed with…”, 40 (90.9%) of our 44 participants selected a careful history, of which 31 (70.5%) also selected physical examination and/or investigations, and 17 (39%) also selected a psychiatric history. Thus, only 23 (52.3%) participants selected the correct combination of a careful history without a psychiatric history, with or without physical examination or investigations. (Table 6).

Respondents were then presented with a number of propositions regarding clinical features required for a diagnosis of ME to be made. Some of these propositions were true and some were false. Thus, 38 participants (86.3%; 95% confidence interval: 73.3–93.6%) believed, erroneously, that six months of fatigue was necessary for diagnosis. A significant 39% of respondents did not realise that post exertional malaise is an essential requirement for the diagnosis of ME. Psychiatric features are not part of the diagnosis, but only 24 of 44 respondents recognised this (54.5%; 95% confidence interval 40.1–68.3). A total of 17 participants selected psychiatric symptoms, signs of anxiety or depression, or both, and three participants failed to select any answer. Only six of 44 respondents selected the correct combination of features (i.e., post exertional malaise and symptoms from multiple systems, without psychiatric features (i.e., 13.6%; 95% confidence interval 6.4–26.7). The results are detailed in Table 7.

### 3.5. Disability, Impact and Clinical Manifestations of ME

When asked about the level of disability suffered by ME patients, 64% of respondents under-estimated the level of disability compared to other common or serious illnesses (Table 8). Only 36% of respondents correctly recognised that ME patients can be as disabled as patients with all seven of the other conditions named. These are multiple sclerosis, cancer, advanced HIV, chronic respiratory disease, end stage renal failure, heart failure and a broken leg. In total, 45% of respondents over-estimated the ability of ME patients to stay in work (Table 5). The vast majority (97.7%, 43 out of 44, 95% confidence interval 88.2–99.6%) did, however, recognise that children with ME can miss long periods of school (Table 4). The majority of respondents (79.6%, 35 out of 44, 95% confidence interval 65.5–88.9%) indicated that ME is painful but only a quarter of respondents (25.0%, 11 out of 44, 95% confidence interval 14.6–39.4%) knew that ME can kill (Table 5).

Of our respondents, 70% did not realise the breadth of manifestations and symptoms of ME (Table 5 and Table 9). Seven body systems very commonly affected in ME were listed, and only 30% of respondents indicated that ME can affect all seven body systems, i.e., the nervous system, the cardiovascular system, the endocrine system, the musculoskeletal system, the gastrointestinal system, the immune system and cellular metabolism. These results are summarised in Table 9 below:

### 3.6. Treatment

Almost all (98%) respondents believed that graded exercise therapy (GET) is a suitable treatment for ME. In addition, 61% believed that cognitive behavioural therapy (CBT), designed to assist patients to rethink their illness attributions and abandon the sick role, is also a suitable treatment. These results are summarised in Table 10.

### 3.7. Interest in Further Education on ME

The response to this was very positive. Participants were asked to respond to the statement: “After today’s introductory lecture, I would like further more in-depth teaching on Myalgic Encephalomyelitis.” A total of 36 doctors answered this question. The lower response rate may relate to having had to wait until after they had had the lecture before answering. Of those who responded, 20 said Yes, 3 said No, and 13 were Neutral. Therefore, only a very small minority (8%) did not want further teaching on ME. Over half of the respondents (56%) would welcome further education on ME, and the rest (36%) are presumably amenable to it, making a total of 92% who would be amenable to further education on ME. These results are summarised in Table 11.

### 3.8. Summary of Results

Overall, there was little knowledge of definitions of ME, or of its clinical manifestations and impact, and equally little knowledge of appropriate management of the condition, with the consequence that patients with ME were likely to have imposed on them treatment that is at best ineffective and at worst damaging, like graded exercise therapy. Diagnosis was equally problematic, with little understanding of required clinical features, in particular the essential symptom of post exertional malaise.

The effect of all this ignorance is to put patients at risk, but a saving grace is the very positive response of participants to the prospect of further education on ME.

This audit study captures baseline data, which sadly confirms patients’ perception that their doctors know little about ME and that many do not even believe it is real. By measuring participants’ responses against the reasonable expectation that all participants should get all answers correct, it enables us to highlight errors in basic fundamental understanding, such as the misconception that ME is partly or wholly psychological or psychosomatic. It also enables the highlighting of large deficiencies in education and clinical knowledge on ME, as well as dangerous prevailing ideas on treatment.

## 4. Discussion

### 4.1. Prior Teaching, Experience and Confidence Level

A minority of respondents had had formal teaching on ME, though most had had some experience of ME patients. Despite this, few knew how to diagnose ME, and nearly all lacked confidence in dealing with ME patients.

The majority of participants (82%) believed that ME is at least in part psychological, and it is a matter of concern that 91% of respondents who had had teaching or experience of ME thought this, when only 50% of those without such experience thought so. This places a considerable question mark over the content of such teaching and experience, since those who had received it more frequently expressed erroneous views about ME than those who had not.

It is also of particular note that doctors who expressed confidence in diagnosing ME or in dealing with ME patients were universally wrong in their understanding of the nature of ME, its diagnostic criteria, and its treatment. All six of them (100%) thought ME was at least in part psychological/psychosomatic, failed to select the right combination of diagnostic features, and thought ME could be treated with extremely hazardous graded exercise therapy.

### 4.2. Making the Diagnosis

Myalgic encephalomyelitis is mainly diagnosed with a careful and thorough history. Physical examination and appropriate investigations are performed to rule out other pathology, but the diagnosis is made on the presence of post exertional malaise (PEM) and other symptoms, as identified in the history. While certain physical signs can be present, such as orthostatic changes in blood pressure or heart rate, pallor, and a multitude of neurological signs including tremor, incoordination, ataxia, photophobia, muscle weakness, fatiguability, fasciculations and myopathic facies, they are, like everything else in ME, variable and fluctuating.

On diagnostic criteria, 38 participants (86.3%; 95% confidence interval: 73.3–93.6%) believed six months of fatigue is necessary for diagnosis. This is contrary to the MYALGIC ENCEPHALOMYELITIS—Adult and Paediatric: International Consensus Primer for Medical Practitioners, which allows one to make a positive diagnosis based on symptom constellation, without having to wait six months [16]. This is important as it allows timely diagnosis and management. Diagnostic delay and lack of crucial medical advice in the early part of the illness frequently results in significant harm and increased severity of illness.

A total of 39% of respondents incorrectly believed that psychiatric symptoms, or signs of anxiety or depression, were necessary for a diagnosis of ME, in line with the misconception that ME is a psychological or psychosomatic problem. None of the respondents were in fact psychiatrists, psychologists or psychotherapists. These doctors could misdiagnose depression or other mental health problems as ME, depriving patients of necessary treatment. They could also miss the diagnosis of ME, depriving patients of crucial recognition, medical advice and support. Of course, where ME and depression coexist, both need to be recognised and appropriately managed. It should be noted that comorbid depression is as common in other chronic diseases such as multiple sclerosis as it is in ME [17].

The same proportion did not realise that an essential requirement for diagnosis is post exertional malaise, which is an exacerbation of the symptoms of ME/CFS after exertion, which may be physical or cognitive [16,18]. It is recognised as the defining characteristic of ME/CFS [19], can persist for prolonged periods [20], and is unrelieved by sleep or rest [21]. These doctors could erroneously diagnose ME while missing other pathologies. Only 13.6% of participants chose the correct combination of post exertional malaise and symptoms from multiple systems, without psychiatric features, as being necessary to make the diagnosis.

### 4.3. Clinical Understanding

Most participants responded correctly to a series of propositions on the general epidemiology of ME, and nearly all respondents recognised that children with ME can miss long periods of school. However, it is a matter of concern that around a third of respondents considered the statement “children with ME miss school because their parents support their sick role and this should be discouraged” to be correct. ME/CFS is the single most common cause of long-term school absence for medical reasons in England [22], and this has been shown to be due to physical incapacity rather than anxiety [23]. Given the high incidence of unjustified child protection and safeguarding proceedings instigated against families of children with ME, often with disastrous consequences to the health of these children, this misconception is of grave concern [24].

On the overall categorization of ME, most respondents thought that ME belonged in the class of illness called medically unexplained symptoms. This is an umbrella term that encompasses many conditions once thought to be “functional”, or without a pathological basis, and for which psychological treatments were advised [25]. However, the underlying pathology is steadily being elucidated, so the condition can no longer be regarded as being medically unexplained [26].

There were also considerable misapprehensions among the participants regarding the level of disability suffered by ME patients, with approximately two-thirds of all respondents under-estimating the level of disability among people with ME, compared to other common or serious illnesses. Only just over a third of participants correctly recognised that ME patients can be as disabled as patients with all seven of the other conditions named. These are multiple sclerosis, cancer, advanced HIV, chronic respiratory disease, end stage renal failure, heart failure and a broken leg. All these conditions have previously been identified in the literature or described by expert clinicians as having comparable levels of disability to ME, both in adults [18,27,28,29] and in children [30,31,32].

Similarly, nearly half of the respondents over-estimated the ability of ME patients to stay in work, even though research indicates that loss of employment among people with ME/CFS is widespread. A Spanish community-based study found that 63% of ME/CFS patients were unable to work [33], while the comparable percentage in a large UK study, using data from the UK CFS/ME National Outcomes Database, was 50.1% [34]. This British study found that 998 (50.1%) of 1991 patients had lost employment because of illness. Extrapolation suggested the impact of ME/CFS on employment was responsible for UK annual productivity costs of £102.2 million (range £75.5–£128.9 million) [23]. Another Spanish report from the same research group found that 636 of 1116 people with ME/CFS were unemployed (58.6%) [35], while a Norwegian study of hospital patients [36] found that 43 (45%) of 92 were unemployed. Vink and Vink-Niese in a wide-ranging review of the literature on employment in ME/CFS reported both these studies. They also reported a series of studies by national patient organisations that came to similar conclusions, and additionally demonstrated that where patients were able to continue to work, most had to make adjustments to the nature and duration of the work that they undertook [37].

Most participants appreciated that ME is painful. However, only 25% knew that ME can kill, though research indicates increased mortality from cardiovascular disease, cancer and suicide [38,39], the latter being particularly tragic [40,41]. A recent paper has pointed out that there is a considerable risk to life from malnutrition among patients with very severe ME [42]. About two-thirds of participants did not appreciate the wide range of symptoms occurring in ME patients (Table 5 and Table 9). Seven body systems very commonly affected in ME were listed, and only 30% of respondents indicated that ME can affect all seven body systems (see Table 9). These are the nervous system, the cardiovascular system, the endocrine system, the musculoskeletal system, the gastrointestinal system, the immune system and cellular metabolism [20]. The International Consensus Panel made clear the multi-system nature of the condition in 2012 [16], and this was reiterated in the IACFS/ME (International Association for CFS/ME) Primer for Clinical Practitioners in 2014 [19] and the Institute of Medicine case definition of 2015 [18]. This is applicable to children and adolescents [21] as well as adults.

### 4.4. Hazardous Treatments

The responses regarding treatment were a matter of great concern, with nearly all participants (98%) believing that graded exercise therapy (GET) is a suitable treatment for ME (Table 10), while 61% believed that cognitive behavioural therapy (CBT), designed to help patients get out of the sick role and to rethink their illness beliefs, is also an appropriate treatment. It is salutary to reflect on why such misconceptions have become so widespread. Much of this may have been shaped by previous research on ME, particularly that promoting the cognitive-behavioural model of ME/CFS. Thus, one study concluded that behavioural, cognitive and affective factors had a role in prolonging fatigue and that therefore these factors should be the focus of treatment [43], but later work concluded that this model lacked credibility as it had inadequate supporting evidence and did not address the increasing evidence of pathophysiological changes in ME/CFS [44].

As outlined above, ME is a serious and debilitating multi-system neuro-immune condition. As such, CBT, attempting to convince patients that they are not actually sick, is no more a useful treatment than it is for cancer [45,46]. Instead, by convincing patients that they are not ill, it is likely to cause harm, for patients who over-exert themselves may suffer a deterioration in their illness. Even without the behavioural effects, just travelling to and sitting through unhelpful CBT sessions can be harmful to ME patients, whose energy is in short supply and who already struggle to manage minimum essential daily activities. Patient evidence suggest adverse outcomes occur in 20% of cases treated with CBT [47].

Many of the participants (98%) believed that graded exercise therapy (GET) was a suitable treatment for ME, perhaps not a surprise given that NICE UK included it as a recommended treatment in 2007. However, many doctors may not be aware of how unpopular this treatment is among ME patients [48], or that it can lead to worsening of symptoms for some patients with ME, and there is in any event increasing evidence that such treatment is ineffective and can be damaging in patients of all levels of severity [19]. The evidence base for GET use has revealed that exercise therapy is not an effective treatment for ME. Reanalysis of the largest GET trial, the PACE trial, revealed recovery rates close to just 10% (little above natural recovery rates), rather than the 22% recovery rate reported by the PACE trial authors [49]. Adverse effects in the trial were dismissed as a consequence of inappropriate implementation by inexperienced practitioners [45]. A 2019 Cochrane review considered eight reports on the use of exercise therapy on ME in adults and concluded that such treatment probably had a positive effect on fatigue [50]. However, a subsequent reanalysis found that this analysis was flawed due to the non-reporting of harms in the reports initially studied, and that in fact GET appeared to not only be ineffective but also unsafe [47].

Similarly, a 2011 review of eight surveys found that 51% of survey respondents had reported that GET had made their health worse [51]. An analysis of primary and secondary surveys found that 54–74% of patients responded negatively to GET [52]. The UK ME Association reported this finding, and advised that GET should play no part in activity management advice in ME. They also recommended that CBT, which also impacted negatively on outcomes, should be avoided in ME/CFS [53]. An American report by experienced clinicians concluded that not only did GET fail to improve function, but that it could provoke the hallmark ME symptom of post exertional malaise (PEM) [48]. CBT, similarly, was found to be of benefit to only 8–35% of patients [48], which supports the earlier view of the authors of the IACFS/ME *Primer for Clinical Practitioners* that the belief that CBT and GET can cure ME “is not supported by post-intervention outcome data” [19].

A report from the Centers for Disease Control and Prevention concluded that patients with ME cannot tolerate vigorous aerobic exercise regimes [54], and the evidence on GET continues to accumulate. A recent survey of the experience of ME patients in Italy, Latvia and the UK found that, while none of the Italian or Latvian participants reported having experienced GET, in the UK out of 70 respondents who had had GET, only 1 (1.4%) reported that it had been effective [55]. For these reasons, of ineffectiveness, distress to patients, and risk of harmful sequelae, the National Academy of Medicine in the US no longer recommends GET for ME [18], and it is noteworthy that the draft guideline from NICE in the UK on ME/CFS recommends that GET, or indeed any therapy based on fixed incremental increases in physical activity or exercise, or any programme founded on the supposition that deconditioning is the cause of ME, should no longer be offered to patients [56].

### 4.5. The Urgent Need and Appetite for Medical Education

The results of this study make a strong case for putting Myalgic Encephalomyelitis into formal medical education in the UK. We would argue that with ME being more than twice as common as multiple sclerosis [4] and as debilitating or worse than most other chronic illnesses such as heart failure or end stage renal disease [18,27,28,29] and being the single greatest cause of long term school absence in children [22], the medical profession cannot afford to be so ignorant, and so misinformed, about ME. This becomes even more evident when considering the hazards of currently favoured therapies, as outlined above, in conjunction with the rising costs of clinical negligence [52]. The costs to the UK economy are also considerable, with direct costs estimated at £3.3 billion per annum to the country [57] and productivity costs at £102.2 million per annum [34].

Doctors need to be able to recognise ME regardless of their specialty, as it has such a wide range of symptoms and presentations. Not only does this audit demonstrate the great and urgent need for medical education on ME, which must be scientifically accurate and up-to-date, responses also demonstrate the appetite for it. More than half the respondents (56%) who answered this question wished to have more in-depth teaching on ME, and a total of 92% were amenable to it. Medical royal colleges and medical schools should take heed.

### 4.6. Strengths and Weaknesses

The main strength of this study is that it is one of the few studies in the United Kingdom to make a formal appraisal of doctors’ knowledge and understanding of myalgic encephalomyelitis. It also conducted an investigation into the beliefs regarding ME of a group of hospital doctors. The weakness of the study is that it was relatively small-scale, ad hoc and may not be representative of all doctors’ views. Furthermore, the small size of the study meant that only relatively large effects could be detected. However, our findings do appear to be consistent with other studies [58,59], and such findings of poor knowledge and negative attitudes appear persistent over decades. These may be linked to how doctors are taught and trained in UK medical schools [59], with both doctors and medical students developing their ideas about ME from lay and informal sources rather than scientific knowledge and evidence on the disease. Although attendance at the training event was mandatory, the participants were self-selected, since returning the survey was not obligatory and participants opted to take part in the survey, which may reflect a self-selection bias. Clearly, future research is needed, with larger samples, the involvement of doctors from different specialties, and the use of a pre-post design in any future training event in order to assess the impact of the event on participants’ knowledge of ME.

## 5. Conclusions and Recommendations

ME suffers from being a Cinderella topic within the medical profession, largely ignored by the research community, as is evidenced by very low levels of institutional research funding over many years [60], as well as by high levels of ignorance and disbelief among doctors. This clinical audit has sought to investigate the beliefs about ME of a group of hospital doctors attending a training event and their knowledge and understanding of the condition. It has demonstrated areas of ignorance so considerable that patients treated on the basis of this would be put very much at risk. Nevertheless, it was encouraging that participants recognised a need for further training and indicated a wish to participate in this. It is strongly recommended that scientifically accurate and up-to-date medical education on ME be made a priority at undergraduate and postgraduate levels. It is also recommended that this audit be repeated following a period of medical education.

## Figures and Tables

**Table 1:** Prior teaching and experience of ME, confidence to diagnose and manage it.

	Number of Respondents	Responding ‘Yes’		95% Confidence Interval
		Number	% Total	
Have received some formal teaching on ME	44	12	37.9	16.3–41.9
Have seen some ME patients	44	31	70.5	55.8–81,8
I know how to diagnose ME	44	5	11.4	5.0–24.0
I feel confident dealing with ME patients	44	3	6.8	2.4–18.2

**Table 2:** Relationship between confidence in diagnosing ME and confidence in managing it.

	I Feel Confident Dealing with ME Patients		Total
I know how to diagnose ME	Yes	No	
Yes	2	3	5
No	1	38	39
Total	3	41	44

*p* (by Fisher’s exact test) = 0.029.

**Table 3:** Effect of previous teaching or experience on understanding of ME.

	Thinks ME Is at Least in Part Psychological	Knows ME Is Physical	Total
Had received teaching on ME or has seen some ME patients	31	0	34 (3 don’t know)
Not had teaching on ME and not seen any ME patients	5	4	10 (1 don’t know)
Total	36	4	44

*p* (by Fisher’s exact test) = 0.0002.

**Table 4:** Responses to propositions regarding the nature and epidemiology of ME.

Proposition	Correct Answer	Number of Respondents	Respondents Giving Correct Answer		95% Confidence Interval (%)
			Number	% Total	
We have national guidelines on ME.	True	44	25	56.8	42.2–70.3
ME is rare.	False	44	31	70.5	55.8–82.8
ME affects more women than men.	True	44	40	90.9	78.8–96.4
ME can affect children.	True	44	36	81.8	68.0–90.4
ME resolves within 6 months.	False	44	41	93.2	81.8–97.7
ME causes chronic disability.	True	44	42	95.5	84.9–98.7
If they do not improve it’s because they are not trying hard enough.	False	44	40	90.9	78.8–96.4
Children with ME can miss long periods of school.	True	44	43	97.7	88.2–99.6
Children with ME miss school because their parents support their sick role and this should be discouraged.	False	44	28	63.6	48.9–76.2

**Table 5:** Respondents’ knowledge of definitions and clinical understanding.

Question	Correct Answer	Number of Respondents	Respondents Giving Correct Answer		95% Confidence Interval (%)
			Number	% Total	
Is ME is a physical illness or psychological?	Physical	44	4	9.1	3.6–21.1
ME belongs in the class of illness called Medically Unexplained Symptoms. True or false?	False	44	12	27.3	16.3–41.9
Myalgic Encephalomyelitis, Chronic Fatigue Syndrome and Post Viral Fatigue Syndrome all mean the same thing. True or false?	False	44	18	18.2	27.7–55.6
ME is painful. True or false?	True	44	35	79.5	65.5–88.9
ME is as disabling as: MS, cancer, advanced HIV, chronic respiratory disease, end stage renal failure, heart failure, broken leg. True or false?	All 7 conditions	44	16	36.4	23.7–51.3
Which of the following body systems can ME affect? Nervous system, cardiovascular system, endocrine system, musculoskeletal system, gastrointestinal system, immune system, cellular metabolism.	All 7 body systems	44	13	29.5	18.2–44.2
What proportion of ME patients is able to work?	Less than half	44	22	50.0	35.8–64.2
ME doesn’t kill. True or false?	False	44	11	25.0	14.6–39.4
Patients need to think positive and build up their strength with exercise or gradually increasing activity. True or false?	False	44	2	4.5	1.2–15.4

**Table 6:** Respondents’ views on diagnostic methods.

	Correct Answer	Number of Respondents	Respondents Making Correct Choice		95% Confidence Interval
			Number	% Total	
ME is mainly diagnosed with: (multiple options allowed)					
Careful history	Yes	44	40	90.9	78.8–96.4
Psychiatric history	No	44	27	61.4	46.6–74.3
Right combination (careful history without psychiatric history)		44	23	52.3	37.9–67.3

**Table 7:** Diagnostic requirements.

**Proposition**	**True or False?**	**Number of Respondents**	**Correct Answer Selected?**		**95% Confidence Interval**
			**Number**	**% Total**	
The diagnosis of ME requires:					
• Fatigue lasting at least 6 months	False	44	3	6.8	2.4–18.2
• Psychiatric symptoms (i)	False	44	30	68.2	53.4–80.9
• Post Exertional Malaise (PEM)	True	44	27	61.4	46.6–74.3
• Symptoms from multiple systems	True	44	31	70.5	55.8–81.8
• Signs of anxiety or depression (ii)	False	44	26	59.1	44.4–72.3
• Physical signs	False	44	28	63.6	48.9–76.2
**Combination**		**Number of Respondents**	**This Combination Selected?**		**95% Confidence Interval**
			**Number**	**% Total**	
Don’t know (i.e., no feature selected)		44	3	6.8	2.4–18.2
Any psychiatric feature-(i) or (ii) selected		44	17	38.6	25.7–53.4
Correct combination (PEM, symptoms from multiple systems, no psychiatric features)		44	6	13.6	6.4–26.7

**Table 8:** The impact of ME—Perceived level of disability.

	Total Respondents	Respondents Selecting:		95% Confidence Interval
		Number	% Total	
Question: Patients with ME can be as disabled as patients with (viz. multiple sclerosis, cancer, advanced HIV, chronic respiratory disease, end stage renal failure, heart failure, broken leg).				
Number of conditions in respect of which ME is regarded as being as disabling or more so:				
0	44	4	9.1	3.6–21.2
1	44	11	25.0	14.6–39.4
2	44	3	6.8	2.4–18.2
3	44	4	9.1	3.6–21.2
4	44	3	6.8	2.4–18.2
5	44	0	0.0	-
6	44	3	6.8	2.4–18.2
All 7 (correct answer)	44	16	36.4	23.8–52.3
<7 (incorrect)	44	28	63.6	48.9–76.2

**Table 9:** The Impact of ME—Perceived extent of involvement of body systems.

	Total Respondents	Respondents Selecting:		95% Confidence Interval
		Number	% Total	
Question: ME can affect… (nervous system, cardiovascular system, endocrine system, musculoskeletal system, gastrointestinal system, immune system, cellular metabolism)				
Number of body systems thought to be capable of being affected by ME:				
0	44	4	9.1	3.6–21.2
1	44	1	2.3	0.4–11.8
2	44	3	6.8	2.4–18.2
3	44	7	15.9	7.9–29.4
4	44	4	9.1	3.6–21.2
5	44	4	9.1	3.6–21.2
6	44	8	18.2	9.5–32.0
All 7 (correct answer)	44	13	29.6	18.2–44.2
<7 (incorrect)	44	31	70.5	55.8–81.8

**Table 10:** Respondents’ opinions regarding specific therapies for ME.

Treatment Options (Not Mutually Exclusive)	Number of Respondents	Respondents Selecting Treatment		95% Confidence Interval
		Number	% Total	
Inappropriate therapies:				
Graded exercise therapy	44	43	97.7	88.2–99.6
Cognitive behaviour therapy	44	27	62.8	47.9–75.6
Any harmful treatment selected (GET or CBT)	44	43	97.7	88.2–99.6
Other therapies:				
Antivirals	44	3	7.0	2.4–18.6
Vitamin supplements	44	7	16.3	8.1–30.0

**Table 11:** Interest in further education on ME.

	Answer Options	Total Respondents	Number of Respondents Selecting Response		95% Confidence Interval (%)
			Number	% Total	
Participants requesting further in-depth teaching on Myalgic Encephalomyelitis	Yes	36	20	55.6	39.6–70.5
	Undecided	36	13	36.1	22.5–52.4
	No	36	3	8.3	2.9–23.6

## Data Availability

Tabulations of the original data are available from the corresponding author.

## Appendix A. Doctors’ Knowledge and Understanding of ME, UK 2018

Myalgic Encephalomyelitis-please base answers on your knowledge before today’s lecture.

**Education on ME, Prior Experience, Confidence**	
I have received formal teaching on ME in:	Undergraduate lectures Yes □ No □
	Undergraduate e-learning or PBL Yes □ No □
	Postgraduate lectures Yes □ No □
I have seen ME patients in:	GP clinics Yes □ No □
	Specialty clinics Yes □ No □ If yes, which _________________________
	In hospital Yes □ No □ (tick no if it was just an item on the GP summary list)
I know how to diagnose ME:	Yes □ No □
I feel confident dealing with ME patients:	Yes □ No □
Knowledge on ME: (tick all that apply)	
ME is a:	psychological/psychosomatic illness □ physical illness □
ME is rare:	Yes □ No □
ME affects more:	Men □ Women □
ME can affect children:	Yes □ No □
ME resolves within 6 months:	Yes □ No □
ME belongs in the class of illness called Medically Unexplained Symptoms.	True □ False □
Myalgic Encephalomyelitis (ME), Chronic Fatigue Syndrome (CFS) and Postviral Fatigue Syndrome (PVFS) all mean the same thing.	True □ False □
ME is mainly diagnosed with:	A careful history □
	A thorough physical examination □
	Investigations □
	A psychiatric history □
The diagnosis of ME requires:	Six months of fatigue □
	Symptoms from multiple systems □
	Psychiatric symptoms □
	Signs of anxiety or depression □
	Post exertional malaise □
	Certain physical signs □
Patients with ME can be as disabled as patients with:	MS □
	Advanced HIV □
	Heart failure □
	Cancer □
	Chronic respiratory disease □
	A broken leg □
	End stage renal failure □
ME doesn’t kill	True □ False □
ME causes chronic disability	True □ False □
ME is painful	True □ False □
Children with ME can miss long periods of school	True □ False □
How many ME patients are able to work?	Most of them □
	About half □
	Less than half □
ME can affect:	The cardiovascular system □
	The musculoskeletal system □
	The nervous system □
	The immune system □
	The endocrine system □
	Cellular metabolism □
	The gastrointestinal system □
ME can be treated with:	Antivirals □
	Graded Exercise Therapy □
	Vitamin supplements □
	CBT to help patients get out of the sick role □
Patients need to think positive and build up their strength with exercise or gradually increasing activity.	True □ False □
If they do not improve it’s because they’re not trying hard enough	True □ False □
Children with ME miss school because their parents support their sick role and this should be discouraged.	True □ False □
We have national guidelines on ME.	True □ False □
After today’s introductory lecture, I would like further more in-depth teaching on Myalgic Encephalomyelitis:	Yes □
	No □
	Neutral □
